# The *Myxococcus xanthus* Spore Cuticula Protein C Is a Fragment of FibA, an Extracellular Metalloprotease Produced Exclusively in Aggregated Cells

**DOI:** 10.1371/journal.pone.0028968

**Published:** 2011-12-12

**Authors:** Bongsoo Lee, Petra Mann, Vidhi Grover, Anke Treuner-Lange, Jörg Kahnt, Penelope I. Higgs

**Affiliations:** 1 Department of Ecophysiology, Max Planck Institute for Terrestrial Microbiology, Marburg, Germany; 2 Institute for Microbiology and Molecular Biology, Justus-Liebig University of Giessen, Giessen, Germany; Center for Cancer Research, National Cancer Institute, United States of America

## Abstract

*Myxococcus xanthus* is a soil bacterium with a complex life cycle involving distinct cell fates, including production of environmentally resistant spores to withstand periods of nutrient limitation. Spores are surrounded by an apparently self-assembling cuticula containing at least Proteins S and C; the gene encoding Protein C is unknown. During analyses of cell heterogeneity in *M. xanthus*, we observed that Protein C accumulated exclusively in cells found in aggregates. Using mass spectrometry analysis of Protein C either isolated from spore cuticula or immunoprecipitated from aggregated cells, we demonstrate that Protein C is actually a proteolytic fragment of the previously identified but functionally elusive zinc metalloprotease, FibA. Subpopulation specific FibA accumulation is not due to transcriptional regulation suggesting post-transcriptional regulation mechanisms mediate its heterogeneous accumulation patterns.

## Introduction

*Myxococcus xanthus* is a Gram-negative soil bacterium which glides across surfaces using a combination of type four pili (T4P)-mediated social (S)-motility and single cell adventurous (A)-motility which is thought to be mediated by focal-adhesion complexes [1]. Predatory swarms of cells obtain nutrients by digesting prey microorganisms or decaying organic material [2]. Under nutrient limited conditions, the cells enter a complex developmental program with at least three distinct cell fates. While the majority of cells lyse [3], [4], some cells aggregate into mounds of approximately 100,000 cells and then, exclusively within these mounds (fruiting bodies), differentiate into environmentally resistant spores [5]. An additional population of cells differentiates into peripheral rods which do not aggregate nor sporulate and remain outside of the fruiting bodies [6]. Thus, there is significant heterogeneity in the developing population and identification of markers for these different cells is of importance in understanding when and how the developmental population segregates into distinct cell fates.

*M. xanthus* spores, which are resistant to desiccation, heat, and sonic disruption, contain a polysaccharide-rich spore coat surrounded by an apparent self-assembling cuticula consisting of at least Protein S and Protein C [7], [8], [9]. Protein S, a member of the beta gamma-crystallin superfamily [10], is not necessary for spore formation or viability and may be instead related to spore adhesiveness in fruiting bodies [11]. Protein C was identified as a prominent ∼31 kDa protein band during denaturing polyacrylamide gel electrophoresis of isolated spore coats [9]. Antisera generated against this excised band demonstrated that Protein C was not produced in vegetative cells, but increased after induction of starvation [9].

Here, we demonstrate that Protein C is produced in a subset of cells that are found in aggregates, under both vegetative and developmental conditions. We determine that Protein C is actually a fragment of FibA, a previously characterized zinc metalloprotease which is primarily localized in the extracellular matrix material (ECM) of the cell [12], [13], [14]. FibA accumulation in aggregated cells appears to be the result of a post-transcriptional regulatory mechanism.

## Results and Discussion

### Protein C displays heterogeneous accumulation

**Figure 1:**
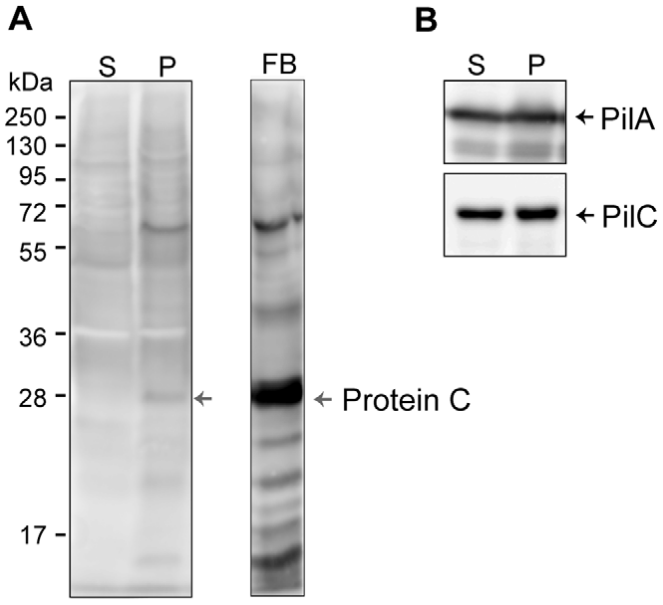
Protein C accumulation is heterogeneous. **A**. Anti-Protein C [9] immunoblot analysis of wild type (*M. xanthus* strain DZ2) cells grown on the surface of a Petri plate in vegetative (CYE) media. Cells were harvested, and cells in aggregates were pelleted at 50 x g for 5 min. Each lane contains lysate from 4.3×10^7^ cells harvested from the supernatant (S) or aggregated cell pellet (P) fractions. FB: Control demonstrating the Protein C accumulation in 4.3×10^7^ cells isolated from fruiting bodies formed after 48 hours of development. B. Anti-PilA [17] (top panel) and anti-PilC [16] (bottom panel) immunoblot of the supernatant (S) or pellet (P) fractions from A. Protein C was detected exclusively in the pellet cell fraction whereas PilA and PilC were equally represented in both cell fractions.

As part of our ongoing analysis of *M. xanthus* population heterogeneity, we employed a low-speed centrifugation assay [6], [15] to separate cells in aggregates from the remaining population which remains in the supernatant. Cells in these two fractions were enumerated, resuspended to equal cell concentration and analyzed by immunoblot with various markers for the alternate cell fates, including anti-sera to Protein C, a previously described component of the spore cuticula produced during developmental conditions [9]. Surprisingly, in addition to detecting the ∼31 kDa Protein C band (grey arrows) in the fruiting body (FB) population of starving cells, we could detect Protein C in cells growing under vegetative conditions, but only in the aggregating cell fraction (Fig. 1A). As a control that we loaded lysates prepared from equal numbers of cells, we probed the same samples with anti-sera to PilC [16] and PilA [17], the inner membrane and pilin components of the T4P motility machinery, respectively. These two proteins were equally represented in both supernatant and aggregating cell fractions (Fig. 1B). Thus, we rationalized that Protein C, for which the corresponding gene is unknown, may play an additional role in *M. xanthus* biology and could represent a marker for a subset of cells in the heterogeneous population.

### Protein C is encoded by Mxan_6106 (*fibA*)

To identify Protein C, we used two different approaches. In the first approach, we isolated Protein C from spores following the original Protein C isolation protocol [9]. Spores were boiled, and the released surface proteins were resolved by denaturing polyacrylamide electrophoresis. The ∼31 kDa Protein C band was excised and subjected to mass spectrometry analysis. The most abundant *M. xanthus* protein identified (with 42 unique peptides and a total ion score of 3506) corresponded to the gene Mxan_6106. In the second approach, we used the anti-Protein C sera to immunoprecipitate the aggregated cell fraction from cells developed under submerged culture for 24 hours. The most abundant *M. xanthus* protein identified (18 unique peptides; total ion score 1799) was also encoded by Mxan_6106.

**Figure 2:**
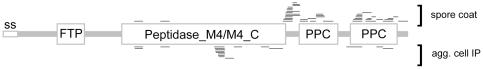
Mass spectrometry maps Protein C to FibA. Domain architecture of the 744 amino acid (aa) FibA preprometalloprotease as predicted by SMART (http://smart.embl-heidelberg.de) [54]. ss: signal sequence (aa 1–24); FTP: Fungalysin/Thermolysin Propeptide PFAM domain (aa 100–149); Peptidase _M4/M4_C: peptidase family M4 and M4-Cterminal PFAM domains (aa 218–518); PPC: Bacterial pre-peptidase C-terminal PFAM domain (aa 544–614 and 638–724). Black lines correspond to the length and sequence position of the peptides identified from mass spectrometry analysis of Protein C isolated from the spore coat (above schematic) or by immune-precipitation of the aggregated cell fraction using anti-Protein C sera (below schematic).

Mxan_6106 was previously characterized as *fibA* (fibril protein A) [13] which is predicted to encode a 79.8 kDa pre-pro-zinc metalloprotease containing a predicted type II secretion sequence, followed by FTP propeptide, peptidase family M4, Peptidase_M4_C, and two pre-peptidase C-terminal (PPC) PFAM domains [13], [18] (Fig. 2). FibA can be detected as ∼ 66 and 31 kDa bands in immunoblot analysis [13], [14] and has been localized in the inner membrane (proposed to be the lipid anchored inactive pro-form) and in the extracellular matrix (ECM; *a.k.a.* fibrils) [14], [19]. 40 out of 42 peptides identified by mass spectrometry analysis of the ∼31 kDa Protein C isolated from spore coats corresponded to the carboxy-terminal region of FibA (aa 515–744) which encompasses the two PPC domains (Fig. 2, lines above schematic). In contrast, the peptides identified from the sample in which the Protein C sera was used for immunoprecipitation of the aggregated cell fraction span amino acids 242–699 which additionally includes most of the peptidase region (Fig. 2, lines below schematic).

**Figure 3:**
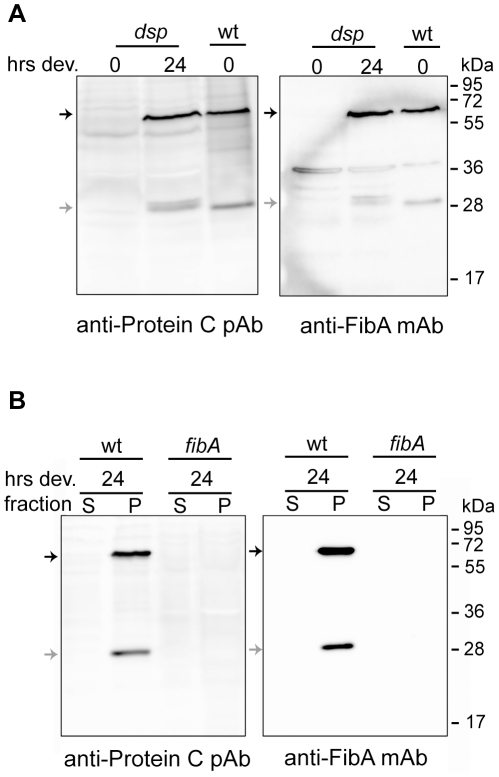
Protein C is the 31 kDa fragment of FibA. **A and B**) Immunoblot analysis using anti-Protein C polyclonal antibodies (pAb) [9] (left) and anti-FibA 2105 monoclonal antibodies (mAb) [13] (right). Black arrows: ∼66 kDa band previously assigned to FibA [13]. Grey arrows: ∼31 kDa band previously assigned to FibA [13] and to Protein C [9]. **A**) Total cell lysates prepared from equal numbers of cells from vegetative cultures (0 hours development) of *dsp* (DK3470) and wild type (DZ2) cells, and *dsp* cultures developing for 24 hours on nutrient-limited CF agar plates. **B**) FibA is present only in the aggregated cell fraction. Wild type (DZ2) and *fibA* (PH1018) cells were developed under submerged culture for 24 hours. Cell lysates were prepared from aggregated cell fractions (P) and supernatant cell fractions (S) as described in Fig. 1.

It was previously shown that FibA is not expressed in *dsp* (aka *dif*) mutants under vegetative conditions; these mutants are defective in ECM production [20], [21], [22], [23]. To ascertain whether the protein C antisera could be specific for FibA, we next performed immunoblot analysis using either the Protein C anti-sera or the FibA monoclonal antibody 2105 [13] on total population cell lysates from vegetative broth culture and cells developed for 24 hours on CF agar plates of strains DZ2 and DK3470 (DK1622 *dsp-1693*) [24] (Fig. 3A). Under vegetative conditions, both antibodies detected identical ∼66 and ∼31 kDa products in wild type but not *dsp* cell lysates. Surprisingly, however, both antisera could detect the two FibA bands in the *dsp* mutant which had been developed for 24 hours, indicating that under starvation conditions, the *dsp* mutation does not prevent accumulation of FibA. To our knowledge, this is the first instance in which the production of FibA in the *dsp* mutant was examined under starvation conditions. We could not isolate an aggregated cell fraction from the *dsp* mutant (data not shown) consistent with ECM being necessary for cell cohesion [25], [26]. Importantly, however, these observations demonstrate that both Protein C antisera and FibA antisera recognize the same products.

To confirm these results, we next generated a disruption in the *fibA* gene in our DZ2 wild type background and performed immunoblot analysis with either the Protein C or FibA antisera on wild type and *fibA*::pPH163 mutant aggregated cell fractions harvested from cells developed under submerged culture for 24 hours (Fig 3B). Both the ∼66 and 31 kDa FibA bands could be detected by both sera only in the aggregated cell fraction of wild type, but not the *fibA*::pPH163 mutant. These results conclusively demonstrated that Protein C is the ∼31 kDa FibA fragment. This fragment was previously assigned as a C-terminal portion of FibA which includes the PPC repeats and which is localized in the ECM of developing cells [14], [27]. PPC repeats, which are often associated with extracellular proteases, are thought to be cleaved from the mature protease. They have been reported to be necessary for appropriate localization, activity, and/or stability of the protease [28], [29], [30], [31]. However, it is not clear why this region should be found as a dominant protein in the spore cuticula. A clue comes from the observation that PPC domains have also been reported to share putative structural and functional features with PKD (polycystic kidney disease) domains [32]. Interestingly, the surface layer protein (SLP) of the archaeon *Methanosarcina mazei*, which is thought to be involved in cell-cell attachment, contains the PKD fold [33]. These observations raise the intriguing hypothesis that the PPC containing region of FibA is recycled to form a layer during sporulation.

Our results also indicated that FibA displays a previously unrecognized cell-type specific accumulation pattern because it is found exclusively in cells in aggregates. This cell fraction is also enriched for ECM production (Lee and Higgs, unpublished data). An exact role for FibA is unknown, but under starvation conditions and together with the ECM and the Dif chemosensory locus [34], it is necessary for appropriate chemotactic responses towards dilauroyl phosphatidyl ethanolamine (PE) lipid species [13]. Dilauroyl PE appears to be a functional analog for PE containing the fatty acid 16:1ω5 [35] which is enriched in *M. xanthus* during development [36]. Thus, it has been proposed that FibA/ECM-dependent chemotaxis plays a role in mediating self-recognition during fruiting body formation [37]. Our observation that FibA is localized exclusively in the aggregated cell fraction is consistent with these observations. However, the response and adaption to PE has been measured primarily assays of isolated single cells [38]; our results demonstrating FibA accumulates nearly exclusively in the aggregated cell fraction (*i.e.* where cells are in intimate contact) suggests that the FibA-dependent response to PE in groups of cells should be investigated.

### FibA plays a minor role in sporulation

Since our results indicate that Protein C is a fragment of FibA, we more closely examined whether it was necessary for spore production and viability. We analyzed both the wild type and *fibA*::pPH163 mutant for developmental phenotype under either strict starvation submerged culture conditions or on nutrient-limited CF agar plates. Under submerged culture, no detectable difference in timing or morphology of fruiting bodies was detected (data not shown). However, analysis of the production of heat and sonication resistant spores demonstrated that the *fibA*::pPH163 mutant was both delayed and less efficient in sporulation relative to wild type, ultimately producing 83±9% of the wild type spores isolated at 120 hours of development (Table 1). Furthermore, the *fibA*::pPH163 spores germinated at a rate of 70% of the wild type spores (Table 1). When both strains were instead developed on CF agar plates, both the timing of fruiting body production (data not shown), sporulation, and germination efficiency was similar to wild type (Table 1). It was previously demonstrated that in the *M. xanthus* DK1622 wild type background analyzed on strict starvation TPM agar and at high cell densities, *fibA* mutants produced disorganized fruiting bodies but displayed no significant sporulation defect [13]. Together, these results suggest that the *fibA* sporulation phenotype appears to depend on nutrient levels and/or characteristics of the surface on which the cells are developing. Thus, although Protein C (*i.e.* the ∼31 kDa C-terminal fragment of FibA) is a dominant component of the spore cuticula [9], it is not strictly required for production of resistant viable spores. Similar observations were made for Protein S [11], suggesting the spore cuticula is not, in general, necessary for resistance or viability of spores. Instead, the cuticula may be related to packaging of spores in fruiting bodies since both Protein S and C are produced during fruiting body formation (prior to sporulation) [9], [39] and are not produced during chemical-induction of sporulation [40] in which fruiting body formation is by-passed [41], [42].

**Table 1:** Sporulation efficiencies of wild type and *fibA* cells developed in submerged culture or on CF agar.

		number of spores produced (%)^a^			
conditions	strain	48 hours	72 hours	120 hours	germination rate (%)^b^
subm. culture	wt	1.4 ±0.3×10^7^ (27± 6)	3.0±0.1×10^7^ (58±3)	5.2±0.6×10^7^ (100±12)	33±5% (100)
	*fibA*	5.1 ±0.9×10^6^ (10± 2)	1.9±0.3×10^7^ (38±6)	4.3±0.5×10^7^ (83±9)	23±3% (70)
CF agar	wt	ND^c^	2.5±0.2×10^7^ (51±4)	4.9±0.3×10^7^ (100±7)	26±5% (100)
	*fibA*	ND^c^	2.6±0.5×10^7^ (54±11)	4.7±0.5×10^7^ (96±11)	26±6% (100)

a percent of wild type spores produced at 120 hours of development.

b percent of wild type germinating spores.

c none detected; <1×10^5^ spores per spot (<0.21% of wild type).

### FibA accumulation is not due to differences in transcriptional regulation

Tracking individual cell fates in heterogeneous cell populations is most readily achieved by generation of cell-fate specific promoter fusions to fluorescent proteins [43]. To determine whether the accumulation of FibA specifically in the aggregated cell fraction was due to transcriptional regulation, we generated a constructs (pPH161 and pPH162) bearing the putative promoter region of *fibA* (575 bp upstream and including the *fibA* start codon; P_fibA_) fused to the second codon of the gene encoding either the fluorescent reporter protein, mCherry (P_fibA_-mCherry) or green fluorescent protein (P_fibA_-gfp), respectively, and inserted either of these vectors in the *M. xanthus* Mx8 phage attachment (*attB*) site. Cells bearing either of these constructs [strain PH1019 (DZ2 *attB*::pPH161) or strain PH1020 (DZ2 attB::pPH160)] were induced to develop under submerged culture conditions and compared to the wild type. Unexpectedly, both the P_fibA_-mCherry or -gfp expressing strains displayed a stronger sporulation phenotype producing only 38±9% spores of wild type spores at 120 hours of development which is ∼50% fewer spores than the *fibA*::pPH163 insertion mutant (compare to Table 1). This phenotype did not result from inappropriate production of FibA, because FibA was present exclusively in the aggregated cell fraction and at similar levels as in the wild type (data not shown).

**Figure 4:**
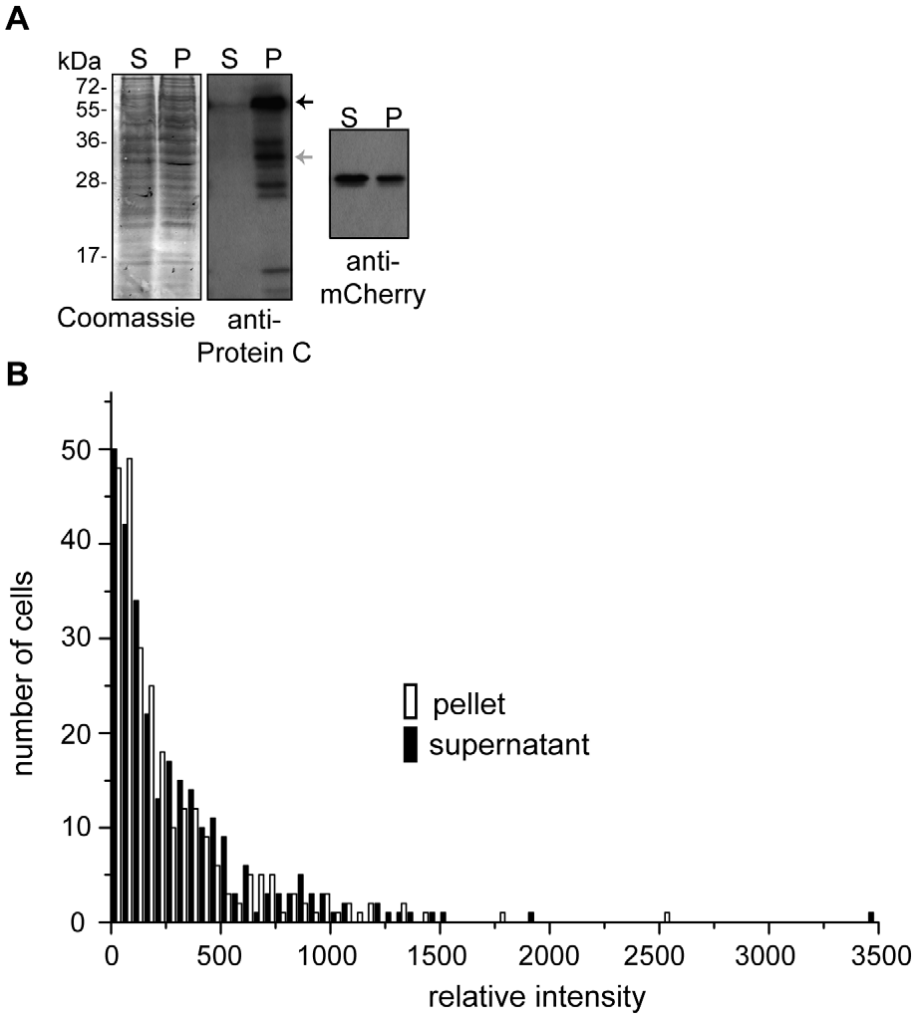
The *fibA* promoter region is active in both cell fractions. **A**) Coomassie stain (left), and anti-Protein C (*aka* FibA) (middle), or anti-mCherry (right) immunoblot analysis of aggregated (P) and supernatant (S) cell fractions harvested from strain PH1019 (DZ2 *attB*::P_fibA_-mCherry) developed for 24 hours under submerged culture. Supernatant and aggregated cell lysates were prepared from equal numbers of cells. Black and grey arrows indicate the ∼66 kDa and ∼31 kDa bands previously attributed to FibA and FibA/Protein C, respectively. **B**. Distribution of individual cell mCherry fluorescence intensities recorded from the samples above. Background subtracted intensity measurements of ≥250 cells from each fraction were recorded. The distribution of intensity measurements (bin size 50 relative intensity values) is displayed as a histogram for the aggregated (pelleted fraction) and supernatant cell fractions as indicated. Histograms were generated using Origin (ver. 6.1) data analysis and graphing software (Northampton, MA, USA). **A and B**. Results from one assay are shown, but triplicate biological repetitions produced identical results.

Given that the strain bearing the P_fibA_-mCherry construct still produced FibA exclusively in the aggregated cells, we proceeded to use this strain to examine whether the *fibA* promoter was equally active in both the aggregated and supernatant population of cells. Cells were developed in triplicate biological replicates under submerged culture for 24 hours, separated into aggregated and supernatant fractions, and lysates prepared from equal numbers of cells in each fraction were subjected to both anti-mCherry or anti-Protein C (FibA) immunoblot (Fig. 4A). FibA was detected nearly exclusively in the aggregated cell fraction, but mCherry was detected nearly equally (relative intensity ratio aggregated / supernatant of 0.97±0.02) in both cell fractions suggesting that in average, the putative *fibA* promoter was similarly active in both populations. To examine the single cell mCherry accumulation in the two populations, we examined dispersed cells from the two fractions under a fluorescence microscope and quantified the fluorescence intensity of single background-subtracted cells (n≥250). These results indicated that the population average aggregated/supernatant mCherry fluorescence ratio was 0.87±0.09, which is similar to the results from the immunoblot analyses. The single cell mCherry fluorescence variation in these two populations was slightly different with more cells in the supernatant population fluorescing with higher intensity (Fig. 4B). No significant fluorescence could be detected in the wild type cells lacking the reporter (data not shown). Together, these results suggest that accumulation of FibA in the aggregated cell fraction is not due to increased promoter activity (transcription) in the aggregated cell population and suggest that *fibA*/FibA is likely post-transcriptionally regulated. Interestingly, a translation attenuator is predicted [44] for *fibA* since both the predicted AGGAGG ribosome binding site and ATG start codon are predicted to be sequestered in a stable stem-loop structure. These results demonstrate a previously unknown level of complexity involved in the mysterious role of FibA in *M. xanthus* lipid chemotaxis, fruiting body formation and sporulation.

## Materials and Methods

### Growth and development

*M. xanthus* strains were grown vegetatively at 32°C on CYE agar plates [1% Casitone, 0.5% Yeast extract, 10 mM 3-(N-morpholino)propanesulfonic acid (MOPS) pH 7.6, 4 mM MgSO_4_, 1.5% agar] or in CYE broth (CYE lacking agar) with shaking at 220 rpm. Plates were supplemented with 100 µg ml^−1^ kanamycin, where necessary. *E. coli* cells were grown under standard laboratory conditions in Luria-Bertani (LB) broth supplemented with 50 µg ml^−1^ kanamycin where necessary [45].

Development was assayed under submerged culture as described in [46]. Briefly, overnight vegetative broth cultures were diluted to an optical density at 550 nm (OD_550_) of 0.035 in fresh media. For developmental phenotype and sporulation assays, 0.5 ml of diluted cells was added per well to 24 well tissue culture plates and incubated at 32°C for 24 hours. For population heterogeneity assays, 16 ml diluted cells was added to 9 cm Petri plates and incubated at 32°C for 24 hours. To initiate the developmental program, CYE media was replaced by an equivalent volume of MMC starvation media (10 mM MOPS pH 7.6, 2 mM CaCl_2_, 4 mM MgSO_4_) and plates were incubated at 32°C for the respective times indicated. For analysis of development on CF agar plates, cells were grown to mid-log in CYE broth, washed and resuspended to 0.35 OD_550_ in MMC starvation media and 10 µl cells were spotted on CF plates (0.15% Casitone, 0.2% sodium citrate, 0.1% sodium pyruvate, 0.02% (NH_4_)_2_SO_4_, 10 mM MOPS pH 7.6, 8 mM MgSO_4_, 1 mM KH_2_PO_4_, 1.5% agar) and incubated at 32°C. Developmental phenotypes were recorded with a Leica MZ8 stereomicroscope and attached Leica DFC320 camera.

Sporulation efficiencies were determined by harvesting triplicate wells (submerged culture) or spots (CF plates) into 0.5 ml sterile water. Heat and sonication resistant spores were enumerated as described previously [15]. Spore viability (germination efficiency) was assayed as described previously [15]. Briefly, spores harvested from triplicate biological samples were serially diluted in sterile water, suspended in molten CYE containing 1% agar, poured onto CYE plates, and colonies arising by 14 days incubation at 32°C were enumerated. Germination efficiency was calculated as the number of colonies per number of spores added to media.

### Strain construction

Strain PH1018 (DZ2 *fibA*::pPH163) was generated by homologous recombination of pPH163 into *fibA* using previously described protocols [46]. pPH163 contains *fibA* codons 19–231 cloned into the EcoRI and BamHI sites of vector pBJ114 [47]. Plasmid integration into the *fibA* gene (Mxan_6106) was confirmed by PCR using primers specific to the plasmid and *fibA* genetic region. Three independent clones were tested for consistent developmental phenotype.

PH1019 (DZ2 *attB*::P_fibA_-mCherry) and PH1020 (DZ2 *attB*::P_fibA_-gfp) was generated by site-specific recombination of pPH161 and pPH160, respectively, into the *M. xanthus* wild type strain DZ2 Mx8 phage attachment (*attB*) site [48]. Integration in the *attB* locus was selected and confirmed as described previously [48]. Three independent clones were tested for consistent phenotype. pPH161 and pPH160 were constructed by using an over-lap PCR method [15] to fuse the putative promoter region of *fibA* (575 bp upstream and including the *fibA* ATG start codon; P_fibA_) to the second codon of the gene encoding either the fluorescent reporter protein, mCherry or green fluorescent protein, respectively [49]. The resulting fusion amplicons were cloned into the EcoRI and HindIII sites of pSL8 [42] which contains the Mx8 *attP* phage attachment sequence and integration machinery.

### Population fractionation

To separate aggregated cells from the rest of the population, cells grown in submerged culture format on 9 cm Petri dishes were harvested by repeated pipetting in 20 ml pipets, transferred to 50 ml falcon tubes, and centrifuged at 50 x g (Heraeus Multifuge 1 S-R centrifuge in 75002002 G swinging bucket rotor) for 5 min at RT. Cells in the supernatant were carefully removed and enumerated in a cell counter (Beckman Coulter Multisizer 3) using a 20 µm aperture tube. Cells in the pellet (aggregated cell fraction) was resuspended in an equivalent volume of MMC buffer, and both the supernatant and resuspended aggregated cell fractions were dispersed at 5 m s^−1^ for 45 sec in a FastPrep® 24 cell and tissue homogenizer (MP Biomedicals) at 4°C, and enumerated. Aggregated cells were completely dispersed under these conditions. Control experiments indicated that repeated rounds of dispersal did not reduce cell number in either fraction. For protein lysate preparation, cells from both fractions were pelleted for 4 620 x g for 10 min and resuspended to 4.3×10^6^ cells ul^−1^ in 2 x LSB (0.125 M Tris-HCl pH 6.8, 20% glycerol, 4% SDS, 10% 2 β-mercaptoethanol, 0.02% bromophenol blue), heated at 99°C, and stored at −20°C.

### Immunoblot analyses

10 µl protein lysates containing 4.3 × 10^7^ cells were resolved by sodium dodecyl-sulphate poly-acrylamide electrophoresis (SDS-PAGE) as described previously [46] except 12% polyacrylamide gels were used for all analyses. Proteins were transferred to polyvinyldenedifluoride (PVDF) membrane (Millipore) using the Towbin tank transfer protocol [50]. Immunoblot analyses were performed as described previously [46] using the following antibody dilutions: α-Protein C polyclonal (pAb) at 1∶5 000 [9]; α-PilC pAb at 1∶10 000 [16], anti-PilA pAb at 1∶10 000 [17], α-FibA monoclonal antibody (mAb) 2105 [13] at 1∶1000, or anti-mCherry pAb at 1∶10 000 [51]. Secondary α-rabbit or anti-mouse IgG-horseradish peroxidase (HRP) antibodies (Pierce) were used at 1∶20 000, or 1∶2 500, respectively, and signals were detected with enhanced chemiluminescence substrate (Pierce) followed by exposure to autoradiography film or detected in a LAS-4000 luminescent image analyzer (Fuji). Relative band intensities were determined with ImageJ (NIH). To visualize total proteins transferred, PVDF membranes were stained with membrane stain solution (50% v/v methanol, 7% v/v glacial acetic acid and 0.1 w/v% Coomassie Brilliant Blue dye) and destained in stain solution lacking Coomassie dye.

### Identification of Protein C

Protein C was isolated from spores as per [9]. Briefly, wild type strain DK1622 [52] was grown overnight in CYE broth and resuspended to 4×10^9^ cells ml^−1^ in MMC buffer. 20×10 µl cells were spotted on 10 CF agar plates and incubated for 96 hours at 32°C. Fruiting bodies were scraped from the plates, washed, and then resuspended in 1 ml ice-cold TM buffer (10mM Tris-HCl [pH 7.6], 8 mM MgSO_4_) and non-sporulating cells were lysed by sonic disruption. To purify spores, the suspension was applied onto a sucrose step gradient (3.5 ml each of 7.5, 15, 30, and 60% sucrose in TM buffer) and centrifuged for 60 min at 4000 x g in a Heraeus Multifuge 1 S-R swinging-bucket rotor. Fractions (1 ml) were taken from top to the bottom of the gradient and microscopically examined for the largest number of spores. Fractions 9 and 10 were pooled and re-purified on a second sucrose density gradient as above. Spores from fractions 9 and 10 were pelleted, resuspended in 1 x SDS-loading buffer (2% SDS, 60 mM Tris-HCl [pH 6.8], 10% glycerol, 5 mM EDTA, 100 mM DTT), and boiled for 5 min to release surface proteins. Samples were then resolved on a 15% SDS-polyacrylamide gel and stained with Coomassie (PageBlue™, Fermentas). The ∼30 kDa region of the gel was excised and subject to analysis by mass spectrometry as previously described in detail [53]. Briefly, the excised gel piece was chopped into small pieces, destained with 50% acetonitrile (AcN) containing 20 mM NH_4_HCO_3_, dehydrated with 100% AcN and dried. Gel pieces were rehydrated in 5 mM NH_4_HCO_3_ in 10% AcN containing 0.01 g l^−1^ sequencing-grade modified trypsin (Promega) and incubated for 10 h at 24°C. The resulting peptide mixture was separated into fractions by nanoLC (PepMap100 C-18 RP nanocolumn and UltiMate 3000 liquid chromatography system, Dionex). Each 8 sec-fraction was spotted together with matrix-solution (alpha-cyano-4-hydroxycinnamic acid) on a MALDI-plate. Automated MALDI-TOF-TOF analysis was carried out on a 4800 Proteomics Analyzer (AB Sciex) in positive-ion reflector mode and externally calibrated. MSMS data were searched against an in-house protein database using Mascot embedded into GPS explorer software (AB Sciex).

To immunoprecipitate Protein C from the aggregated cell fraction of cells induced to develop for 24 hours under submerged culture, approximately 2.5×10^9^ cells were solubilized in 150 µl RIPA buffer (10 mM sodium phosphate, pH 7.2, 150 mM NaCl, 1% (v/v) Triton X-100, 1% (w/v) sodium deoxycholate, 0.1 (w/v)% sodium dodecyl sulphate) containing mammalian protease inhibitor cocktail (Sigma). Solubilized cells were incubated for 1 hour at 4°C with 100 µl magnetic Dynabeads-Protein A (Invitrogen) which were coated with anti-Protein C antibodies according to manufacturer's instructions. Beads were recovered, washed, eluted with 0.1V 1 M glycine pH 2.5, and eluate was neutralized with 0.8V 1 M Tris pH 8.0, according to manufacturer's instructions. A mock experiment in which no lysate was added was used as a control for anti-sera proteins. The pH was adjusted to 8 using NH_4_HCO_3_ and incubated with 0.013 g l^−1^ sequencing-grade modified trypsin (Promega) at 30°C for 7 hours. The digest was stopped by addition of acetic acid, and the resulting peptide mixture was analyzed as above.

### P_fibA_-mCherry analyses

To examine the *fibA* promoter activity in populations of aggregated and supernatant cells, triplicate cultures of strains DZ2 and PH1019 were developed for 24 hours under submerged culture in 9 cm Petri plates and aggregated and supernatant fractions were harvested and dispersed as described in the **Population fractionation** section, above. 10 µl samples of each cell fraction from each biological replicate were taken for fluorescence microscopy analysis (below) or for cell enumeration (above). The remaining cells were pelleted, resuspended to 4.3×10^6^ cells µl^−1^ and subject to immunoblot analyses as described above.

For fluorescent microscopy analysis, cells were spotted on agar pads [42] covered with a cover slip and examined under a Zeiss Axio Imager.M1 microscope. mCherry-specific fluorescence signals were detected at 670 nm wavelength, images were recorded with an EM-CCD Cascade 1 K (Photometrics, Tucson) camera. Single cell fluorescence intensities of at least 250 cells from each sample and covering several different fields were measured using MetaMorph ver7.5. The intensity of single cells was determined as the average area intensity of a cell minus the local background fluorescence of an equivalent area. The aggregated∶supernatant ratio of average per cell intensity of the aggregated and supernatant fractions was calculated for each independent biological replicate and average ratio with associated standard deviation was reported. The single cell intensity distribution of the aggregated and supernatant populations was visualized by histogram analysis of intensity measurements in bins of 50 relative intensity units for each biological replicate using Origin (ver. 6.1) data analysis and graphing software (Northampton, MA, USA). Similar results were determined for each replicate, but the distributions are shown for one of the replicates.
